# Evaluation of 11 terrestrial carbon–nitrogen cycle models against observations from two temperate Free-Air CO_2_ Enrichment studies

**DOI:** 10.1111/nph.12697

**Published:** 2014-01-28

**Authors:** Sönke Zaehle, Belinda E Medlyn, Martin G De Kauwe, Anthony P Walker, Michael C Dietze, Thomas Hickler, Yiqi Luo, Ying-Ping Wang, Bassil El-Masri, Peter Thornton, Atul Jain, Shusen Wang, David Warlind, Ensheng Weng, William Parton, Colleen M Iversen, Anne Gallet-Budynek, Heather McCarthy, Adrien Finzi, Paul J Hanson, I Colin Prentice, Ram Oren, Richard J Norby

**Affiliations:** 1 Biogeochemical Integration Department, Max Planck Institute for BiogeochemistryHans-Knöll-Str. 10, D-07745, Jena, Germany; 2 Department of Biological Science, Macquarie UniversitySydney, NSW, 2109, Australia; 3 Oak Ridge National Laboratory, Environmental Sciences Division, Climate Change Science InstituteOak Ridge, TN, 37831, USA; 4 Department of Earth and Environment, Boston UniversityBoston, MA, 02215, USA; 5 Biodiversity and Climate Research Centre (BiK-F), Senckenberg Gesellschaft für NaturforschungD-60325, Frankfurt am Main, Germany; 6 Department of Physical Geography, Goethe UniversityD-60438, Frankfurt am Main, Germany; 7 Department of Microbiology & Plant Biology, University of OklahomaNorman, OK, 73019, USA; 8 CSIRO Marine and Atmospheric ResearchPMB 1, Aspendale, Vic., 3195, Australia; 9 Department of Atmospheric Sciences, University of IllinoisUrbana, IL, 61801, USA; 10 Canada Centre for Mapping and Earth Observation, Natural Resources CanadaOttawa, ON, K1A 0Y7, Canada; 11 Department of Physical Geography and Ecosystem Science, Lund UniversitySE-22362, Lund, Sweden; 12 Department of Ecology and Evolutionary Biology, Princeton UniversityPrinceton, NJ, 08544, USA; 13 Natural Resource Ecology Laboratory, Colorado State UniversityFort Collins, CO, 80523, USA; 14 INRA, UMR1220 TCEMF-33882, Villenave d'Ornon, France; 15 Université de Bordeaux, UMR1220 TCEMF-33175, Gradignan, France; 16 Department of Biology, Boston UniversityBoston, MA, 02215, USA; 17 AXA Chair of Biosphere and Climate Impacts, Department of Life Sciences and Grantham Institute for Climate Change, Imperial College LondonSilwood Park, Ascot, SL5 7PY, UK; 18 Division of Environmental Science & Policy, Nicholas School of the Environment, Duke UniversityDurham, NC, 27708, USA; 19 Department of Forest Ecology & Management, Swedish University of Agricultural Sciences (SLU)SE-901 83, Umeå, Sweden

**Keywords:** carbon (C) storage, CO_2_ fertilization, ecosystem modelling, elevated CO_2_, Free-Air CO_2_ Enrichment (FACE), model evaluation, nitrogen (N) limitation, plant physiology

## Abstract

- We analysed the responses of 11 ecosystem models to elevated atmospheric [CO_2_] (eCO_2_) at two temperate forest ecosystems (Duke and Oak Ridge National Laboratory (ORNL) Free-Air CO_2_ Enrichment (FACE) experiments) to test alternative representations of carbon (C)–nitrogen (N) cycle processes.
- We decomposed the model responses into component processes affecting the response to eCO_2_ and confronted these with observations from the FACE experiments.
- Most of the models reproduced the observed initial enhancement of net primary production (NPP) at both sites, but none was able to simulate both the sustained 10-yr enhancement at Duke and the declining response at ORNL: models generally showed signs of progressive N limitation as a result of lower than observed plant N uptake. Nonetheless, many models showed qualitative agreement with observed component processes. The results suggest that improved representation of above-ground–below-ground interactions and better constraints on plant stoichiometry are important for a predictive understanding of eCO_2_ effects. Improved accuracy of soil organic matter inventories is pivotal to reduce uncertainty in the observed C–N budgets.
- The two FACE experiments are insufficient to fully constrain terrestrial responses to eCO_2_, given the complexity of factors leading to the observed diverging trends, and the consequential inability of the models to explain these trends. Nevertheless, the ecosystem models were able to capture important features of the experiments, lending some support to their projections.

## Introduction

Rising atmospheric [CO_2_] from anthropogenic fossil fuel emissions fertilizes plants (Liebig, 1843; Arrhenius, 1896; Ainsworth & Long, 2005). Biosphere models integrating the effects of [CO_2_] on plant photosynthesis into projections of the global terrestrial carbon (C) balance suggest that elevated atmospheric [CO_2_] (eCO_2_) has caused a large fraction of the land C sequestration during recent decades (Cramer *et al*., 2001; Sitch *et al*., 2013). These models also project that the CO_2_-induced land C sequestration will continue in the future and thereby significantly reduce the accumulation rate of anthropogenic CO_2_ in the atmosphere (Arora *et al*., 2013). However, most of these models do not account for the limited availability of nitrogen (N) for plant uptake and growth in many terrestrial ecosystems (Vitousek & Howarth, 1991), which could attenuate ecosystem C storage in response to eCO_2_: increased C sequestration as a result of eCO_2_ is thought to bind N into less easily available forms of N within a few years after the onset of CO_2_ fertilization, a process referred to as progressive N limitation (PNL; Comins & McMurtrie, 1993; Luo *et al*., 2004). Terrestrial biosphere models that explicitly consider the C–N cycle interaction show that future land C sequestration could be reduced by 50% or more because of N cycle processes (Sokolov *et al*., 2008; Thornton *et al*., 2009; Zaehle *et al*., 2010). However, estimates of the magnitude of this N effect differ strongly among these projections as a result of uncertainty in the representation of key processes determining the strength of the N constraint on land C storage (Zaehle & Dalmonech, 2011).

Free-Air CO_2_ Enrichment (FACE) experiments in N-limited temperate forest ecosystems provide a unique source of empirical evidence for the ecosystem-scale response of the interacting C and N cycle processes to eCO_2_ (Oren *et al*., 2001; Norby *et al*., 2005; Palmroth *et al*., 2006; Finzi *et al*., 2007; Iversen *et al*., 2012). Specific site conditions (young, fast-growing forests established on abandoned soils previously used for agriculture or grazing) and the artificial nature of these experiments (step increase in [CO_2_]) limit the direct application of the measurements to estimate the N constraint on future global net primary production (NPP) and land C uptake. Nonetheless, the fact that the NPP enhancement resulting from experimentally elevated CO_2_ at several temperate forest FACE experiments converged towards a common response size (Norby *et al*., 2005) has led modellers to attempt benchmarking exercises, to evaluate the capacity of terrestrial ecosystem models to simulate average multi-year effects of CO_2_ fertilization (Sitch *et al*., 2008; Piao *et al*., 2013). However, this consistency of response to CO_2_ seen during the initial years has not been maintained as the length of the experiments increased, showing that a single number does not capture the complexities of ecosystem responses to eCO_2_: for instance, the NPP response strongly declined at Oak Ridge National Laboratory (ORNL) FACE towards the end of the experiment, whereas the Duke FACE site showed a sustained eCO_2_ response (McCarthy *et al*., 2010; Norby *et al*., 2010).

In this article, we use 11 ecosystem models to investigate the effects of N availability on the eCO_2_ response of forest productivity and C storage at two forest sites with fairly similar temperate climate (Köppen Cfa), comparable levels of N deposition, but contrasting vegetation: the evergreen, needle-leaved Duke Forest (McCarthy *et al*., 2010) and the deciduous, broad-leaved ORNL Forest (Norby *et al*., 2010) FACE experiments. As the observed ambient forest productivity and N requirement at the beginning of the experiment were comparable at the two sites (see Results), our hypothesis was that the ecosystem models should be able to explain the diverging long-term trends based on the different processes and time scales associated with the different vegetation types.

**Figure 1:**
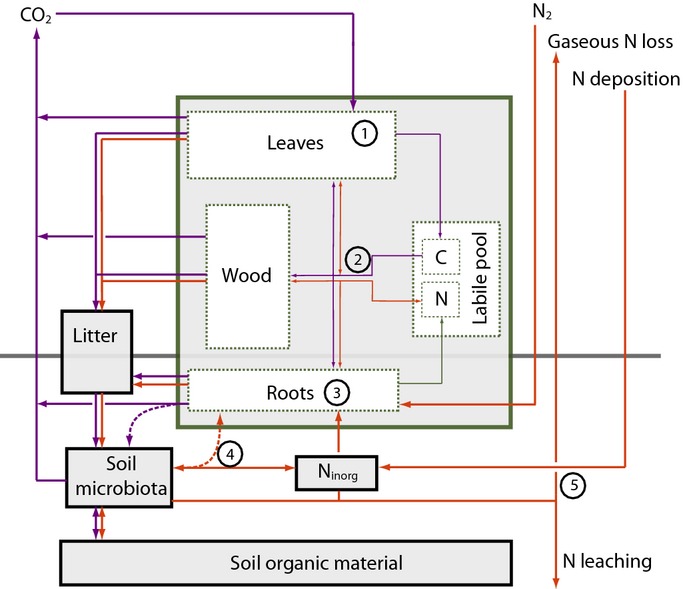
Conceptual diagram of the major nitrogen (N) and carbon (C) flows and stores in a terrestrial ecosystem. Blue arrows denote C fluxes and red arrows N fluxes between major plant compartments (green) and soil pools (black). Numbers 1–5 mark important C–N cycle linkages as described in the Evaluation framework section: 1, N-based gross primary production (GPP_N_): the return of C assimilates per unit canopy N Eqn 1; 2, whole-plant nitrogen-use efficiency (NUE): the total amount of foliar, root and woody production per unit of N taken up by plants; this process depends on the allocation of growth between different plant compartments (e.g. leaves, fine roots and wood) and the C : N stoichiometry of each compartment Eqn 2; 3, plant N uptake (*f*N_up_): the capacity of the plants to take up N from the soil Eqn 4. The plant-available soil N is determined by two factors: 4, net N mineralization (*f*N_min_): the amount of N liberated from organic material through decomposition, which varies with microbial activity and litter quality Eqn 6; and 5, the net ecosystem nitrogen exchange (NNE): based on N inputs from biological N fixation (*f*N_fix_) and atmospheric deposition (*f*N_dep_) and N losses from the ecosystem as a result of leaching to groundwater (*f*N_leach_) and gaseous emission (*f*N_gas_) Eqn 5. As an emergent property, the net amount of C that can be stored in an ecosystem following an increase in CO_2_ depends on the elevated atmospheric [CO_2_] (eCO_2_) effect on the ecosystem's N balance and the whole-ecosystem stoichiometry, which, in turn, depends on the change in the C : N stoichiometry of vegetation and soil, as well as the partitioning of N between vegetation and soil (Rastetter *et al*., 1992).

Our study forms part of a model intercomparison (A. P. Walker *et al*., unpublished) looking at the effect of eCO_2_ on water (De Kauwe *et al*., 2013), C (M. G. De Kauwe *et al*., unpublished) and N cycling. Each of the participating models incorporates the major processes by which the N cycle affects the ecosystem's response to eCO_2_, such as plant N uptake, net N mineralization and the ecosystem N balance, as well as emergent ecosystem properties, such as the N-use efficiency (NUE) of plant production (Fig.1). The representation of these processes varies greatly among models (Table1), illustrating a lack of consensus on the nature of the mechanisms driving these processes. Our objectives in this study were as follows:

1. to understand the eCO_2_ responses predicted by each model for the two sites in terms of their assumptions and representations of C–N cycle processes, and
2. to use experimental observations to constrain these model projections, where possible identifying the mechanisms that are supported vs those that are not.

Given the number and complexity of the C–N processes that determine the observed eCO_2_ responses (Fig.1), and the impracticality of measurement of every relevant C and N flux (e.g. N losses to leaching and gaseous emission) and stock (e.g. changes in organic soil N) with sufficient accuracy, we aimed to identify those process representations that lead to responses qualitatively in agreement with the available C and N cycle observations, rather than identifying the model best fitting the observed NPP responses.

## Materials and Methods

### Experimental sites

The Duke Forest FACE site was located in a loblolly pine (*Pinus taeda* L.) plantation (35.97°N, 79.08°W) established in 1983 in an open woodland partially covered with grass harvested as fodder (McCarthy *et al*., 2007). The soil is relatively nutrient poor, with forest production showing a substantial response to N fertilization (Oren *et al*., 2001; Crous *et al*., 2008; Maier *et al*., 2008), as evidenced from separate N fertilizer experiments in subplots, which were not analysed in the present study. At the start of the Duke FACE experiment in August 1996, trees were 15 yr old and *c*. 14 m tall, with a mean summer leaf area index (LAI) of 3–4 m^2^ m^−2^ (for the dominant pine species). The experiment consisted of three sets of paired plots (pairs of ambient and elevated [CO_2_], each 30 m in diameter) with different levels of tree productivity related to natural variations in soil N availability, affecting ambient NPP, LAI and the C allocation to above- vs below-ground compartments (Finzi *et al*., 2002; Palmroth *et al*., 2006; McCarthy *et al*., 2007). One of each set of plots received continuous enhanced [CO_2_] tracking ambient conditions + 200 μmol mol^−1^.

The ORNL FACE site was located in a sweetgum (*Liquidambar styraciflua* L.) plantation (35.9°N, 84.33°W) established in 1988 on a grassland. The soil at the site had a silty clay–loam texture, and was moderately well drained and slightly acidic (Norby *et al*., 2001; Warren *et al*., 2011). At the start of the experiment, the *c*. 90 trees per 25-m treatment plot were *c*. 12 m tall and in a linear growth phase. The LAI was 5.5 m^2^ m^−2^, and the canopy was no longer expanding (Norby *et al*., 2002). Five treatments plots were established at the site, in two of which exposure to eCO_2_ commenced in April 1998, and continued during the daylight hours of each growing season (April–November). The average daytime [CO_2_] from 1998 to 2008 growing seasons was 547 μmol mol^−1^ in the two CO_2_-enriched plots and 395 μmol mol^−1^ in the three ambient plots.

### Evaluation framework

Our approach to analysing the N cycle dependence of the NPP response to eCO_2_ was to break NPP down into its component processes, thus benefitting from the suite of supplementary observations on these processes provided at each experiment. We investigated how each model represented these individual processes (Table1) and compared model outputs against relevant observations. The key C–N cycle processes controlling the ecosystem response to eCO_2_ (Fig.1) can be grouped into two major categories: Processes affecting NUE (see below), which has both photosynthetic and whole-plant components, and processes affecting N uptake (*f*N_up_), which include the rate of net N mineralization (*f*N_min_), the competitive strength of plant vs soil microorganisms for N assimilation, and the ecosystem's balance of N inputs and losses (net ecosystem N exchange, NNE). All variables used in the following are listed in Table2.

#### N-use efficiency

The change in gross primary production (GPP) with eCO_2_ can be decomposed into the changed C return per unit of N investment into foliage, expressed as GPP per unit leaf N (N-based GPP; GPP_N_) and the change in the amount of leaf N. As the models only reported canopy-integrated values of GPP and foliar N (N_can_), and GPP and autotrophic respiration (*R*_a_) could not be measured directly, we analysed the eCO_2_ effect on the relationship between NPP and N_can_ at the whole-ecosystem level, by analysing the N-based NPP (NPP_N_) as: 

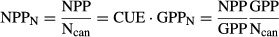
Eqn1

where CUE is the whole-plant C-use efficiency.

NPP is related to the amount of N available for growth by the N requirements set by the relative proportion of biomass growth of the different plant components and their C : N stoichiometry. We decomposed the whole-plant NUE into changes in tissue stoichiometry, changes in tissue allocation and retranslocation as follows: 

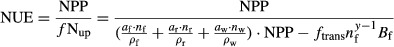
Eqn2

where *a* is the fraction of NPP allocated to foliage (f), fine roots (r) and woody (w) biomass, *n* is the respective tissue N concentration and 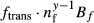 is the amount of N resorbed from the canopy in the previous year. Each of these terms is available from observations, including the amount of N retranslocated, which is calculated from the difference in N concentration between green foliage and leaf litter. Observed *f*N_up_ at ORNL FACE also included an estimate of foliar N uptake from atmospheric N deposition, a process not included in the models, at the rate of 0.6 g N m^−2^ yr^−1^ for both ambient and elevated plots (Norby & Iversen, 2006).

Net changes in vegetation C : N may differ from changes in NUE because N becomes allocated to tissues with different lifetimes. The effect of such changes is reflected in changes in the mean residence time of N in vegetation: 

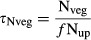
Eqn3 where N_veg_ is the total N in vegetation.

#### Plant N uptake

The plant N uptake (*f*N_up_) can be expressed as the sum of three factors: the rate of net N mineralization into the inorganic N pool from litter and soil organic matter (SOM) decomposition (*f*N_min_), the depletion of the soil inorganic N pool (ΔN_inorg_) and any changes in NNE:



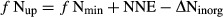
Eqn4a

Changes in NNE depend on inputs from biological fixation (*f*N_fix_) and atmospheric deposition (*f*N_dep_) and losses caused by leaching (*f*N_leach_) and gaseous emission (*f*N_gas_): 

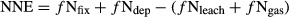
Eqn4b

The rate of net N mineralization (*f*N_min_) can also be separated into two factors: the effect of accumulating soil N during the course of the experiment and changes in the ratio of microbial N immobilization to gross N mineralization as follows: 

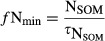
Eqn4c

where N_SOM_ is the size of the decomposing SOM pool, here including the litter layer, and 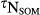 is its apparent turnover time. 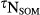 is constant, as long as the ratio of gross N mineralization to immobilization and the allocation of N to SOM pools with different lifetimes do not change. Increasing immobilization as a result of reduced litter quality will increase 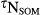, whereas increased gross mineralization from increased microbial N uptake and release will decrease 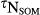. Insufficient observations were available to constrain the change in *f*N_up_ component processes during the course of the experiment (Iversen *et al*., 2011).

#### Ecosystem stoichiometry

The total ecosystem C stored in a forest relates to the total ecosystem N as follows (Rastetter *et al*., 1992): 

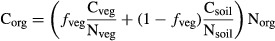
Eqn5where N and C are the N and C pools, respectively, for vegetation (veg), soil (soil) or total organic (org), and *f*_veg_ is the fraction of ecosystem N in vegetation. For the sake of simplicity, litter pools were subsumed to the soil pools.

### Observations

Observed annual changes in C and N cycle parameters were taken from the FACE Data Management System web repository (http://public.ornl.gov/face), as well as published literature, where indicated below. N cycle observations from Duke FACE were only available from 1996 to 2005, and so most of the analyses in this article are focused on this period, although NPP and meteorological forcing data for each treatment plot were available until 2007. The ORNL FACE experiment ran from 1998 to 2009, and data through 2008 were available for this study.

For Duke FACE, standing biomass and biomass production in each plot for three plant compartments (foliage, fine roots and woody biomass, including branches and coarse roots) were taken from McCarthy *et al*. (2010), using the C and N concentration data for each plant compartment reported by Finzi *et al*. (2007) to estimate C and N stocks and fluxes. Plant N requirements and uptake were calculated from these data following Finzi *et al*. (2007). Forest floor and SOM C and N concentrations were obtained from Lichter *et al*. (2008).

For ORNL FACE, standing biomass, annual biomass production, their respective C and N concentrations, as well as inferred N requirements and plant N uptake by plot and plant compartment (foliage, fine roots and woody biomass, including branches and coarse roots), were obtained from Norby *et al*. (2010). Initial and final SOM stocks and their C and N concentrations were obtained from Johnson *et al*. (2004), Jastrow *et al*. (2005) and Iversen *et al*. (2012). Differences in sampling design and soil bulk density measurements prevent an accurate calculation of the change in soil C and N during the course of the experiment (Iversen *et al*., 2012). Comparing the % C and N data in Johnson *et al*. (2004) and Iversen *et al*. (2012), we estimated that 10 ± 21% of the greater C and N stocks in the elevated plots at the end of the experiment (Iversen *et al*., 2012) were a result of eCO_2_, whilst the rest were a result of initial differences among the plots. Combined with the standard errors of the measurements, eCO_2_ led to an increase in SOM to a depth of 90 cm of 160 ± 188 g C m^−2^ and 11.6 ± 24.6 g N m^−2^ between the beginning and end of the experiment.

The data analyses outlined in the Evaluation framework section were made using data by plot and year. For Duke FACE, responses were calculated per plot pair, and reported as the mean and standard error across the three pairs. For ORNL FACE, the analyses were performed with the mean and standard error across the average of the two eCO_2_ plots compared with the average of the three ambient CO_2_ plots.

### Ecosystem models

In this study, we used the same set of 11 process-based ecosystem models as described by A. P. Walker *et al*. (unpublished), encompassing stand (GDAY, DAYCENT, TECO), age/size gap (ED2.1), land surface (CABLE, CLM4, EALCO, ISAM, OCN) and dynamic global vegetation (LPJ-GUESS, SDGVM) models. A detailed account of the major N cycle processes represented in each model is given in Table1. The model simulations covered the time periods representative of the FACE experiments. Meteorological and [CO_2_] data, as well as site history and stand characteristics, were provided in a standardized manner (http://public.ornl.gov/face).

All models (except CABLE and ED2.1) followed a similar protocol to derive the initial soil C and N pools of the sites, which considered the past land use, as well as the historic evolution of atmospheric CO_2_ concentration and N deposition, and site-specific meteorological driver data from during the FACE experiments were used throughout the spin-up. The forest vegetation of the plots was initialized such that the forests had the correct age and structure, as far as considered by the model, at the beginning of the eCO_2_ treatment. Details of the spin-up phase varied among models because of differences in model structure (A. P. Walker *et al*., unpublished). Inherently different assumptions of the models regarding soil C residence times and ecosystem N loss rates, as well as pre-FACE grassland productivity and N fixation, led to a notable spread in the initial amounts of modelled C and N pools, net N mineralization rates and thus NPP, despite the common initialization protocol.

Model outputs were provided at hourly or daily time steps, as appropriate. These outputs contained estimates of the various C, N and water fluxes and pools.

## Results

### Overall response to eCO_2_

Observed ambient NPP and inferred *f*N_up_ at Duke FACE were both slightly larger than at ORNL FACE (Figs3a,b), implying that the whole-plant NUE was similar between the sites (Fig.4) at 121 ± 2 g C g^−1^ N in the ambient plots (1997–2005 mean) for Duke FACE and 129 ± 13 g C g^−1^ N at ORNL. This similarity between sites is in contrast with an earlier study (Finzi *et al*., 2007), because the corrections in biomass estimates by McCarthy *et al*. (2010) resulted in a downward adjustment in the estimate of NUE at Duke Forest.

**Figure 2:**
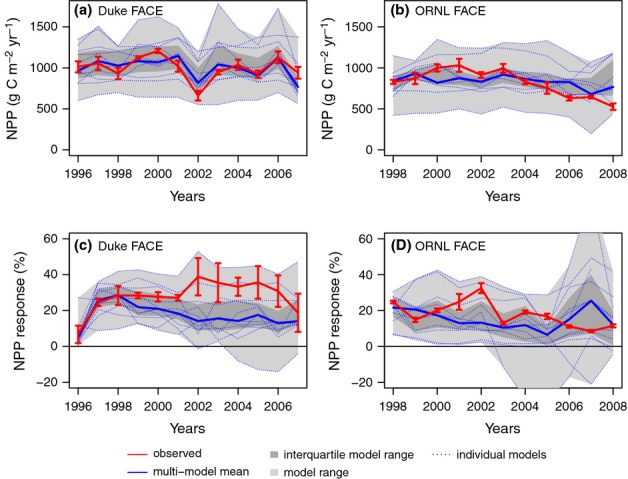
Ambient net primary production (NPP; a, b) and its response to elevated CO_2_ (c, d) at the Duke (a, c) and Oak Ridge National Laboratory (ORNL) (b, d) Free-Air CO_2_ Enrichment (FACE) experiments. The observations are across-plot averages, and the error bars denote ± 1SE.

**Figure 3:**
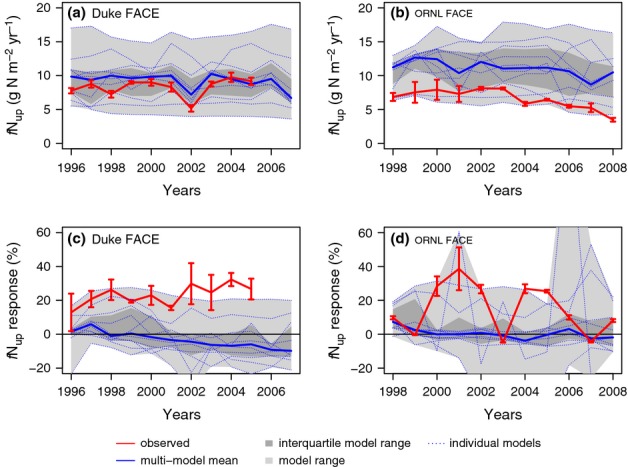
Ambient plant nitrogen (N) uptake (*f*N_up_; a, b) and its response to elevated CO_2_ (c, d) at the Duke (a, c) and Oak Ridge National Laboratory (ORNL) (b, d) Free-Air CO_2_ Enrichment (FACE) experiments. The observations are across-plot averages, and the error bars denote ± 1SE.

**Figure 4:**
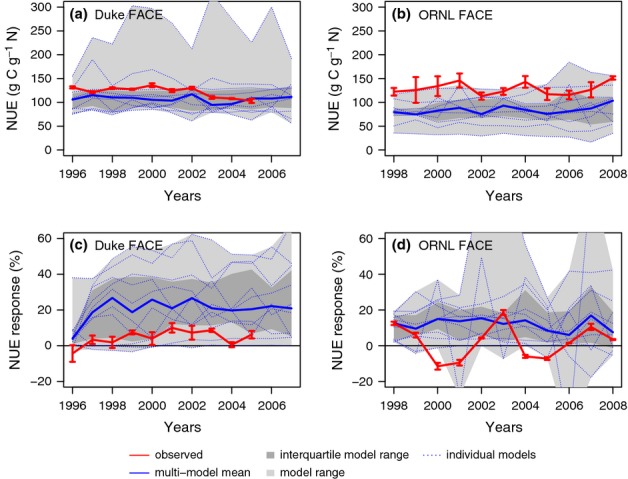
Ambient whole-plant nitrogen-use efficiency (NUE; a, b) and its response to elevated CO_2_ (c, d) at the Duke (a, c) and Oak Ridge National Laboratory (ORNL) (b, d) Free-Air CO_2_ Enrichment (FACE) experiments. The observations are across-plot averages, and the error bars denote ± 1SE.

The interquartile range of the model ensemble included the observed ambient NPP at both sites. However, there was significant spread across the models, resulting to a large extent from different model spin-ups, which led to different levels of N constraints on plant production. Only a few of the models (GDAY, OCN) captured the decline in NPP in the ORNL ambient plots related to declining soil N availability over the course of the experiment (Norby *et al*., 2010; Garten *et al*., 2011). Although the models, on average, matched the inferred, observation-based *f*N_up_ at Duke Forest, they overestimated *f*N_up_ at ORNL (Fig.3). On average, the models slightly underestimated NUE at Duke and more strongly at ORNL FACE (Fig.4). The primary cause for the underestimation was a high bias in the simulation of the fractional (C) allocation to fine roots at both sites (M. G. De Kauwe *et al*., unpublished). At ORNL FACE, this difference was accentuated by higher modelled than observed N concentration of the fine roots (average 1.4% modelled vs 0.7% observed).

**Figure 5:**
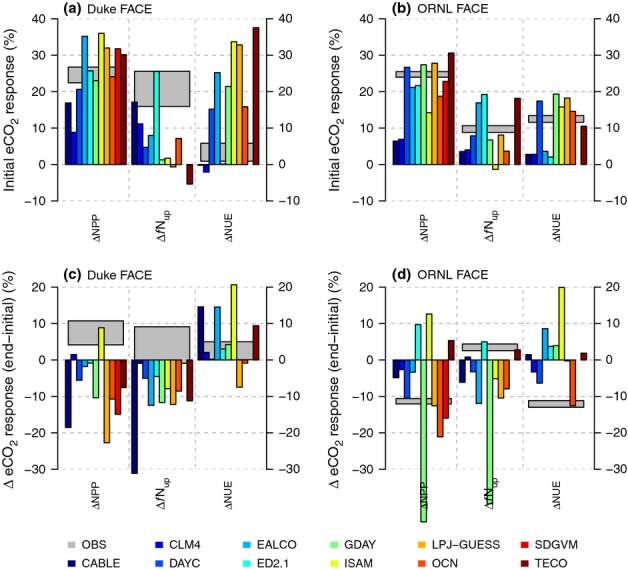
First year response of net primary production (NPP) to elevated atmospheric [CO_2_] (eCO_2_) (a, b) and the change between the first year and the final 5 yr of the experiment (c, d) at the Duke and Oak Ridge National Laboratory (ORNL) Free-Air CO_2_ Enrichment (FACE) sites, respectively, as well as the response of plant nitrogen (N) uptake (*f*N_up_) and whole-plant N-use efficiency (NUE). The grey boxes denote the mean observed eCO_2_ response ± 1SE.

Elevated CO_2_ increased NPP in the initial (first) year of the experiments by 25 ± 9% and 25 ± 1% at Duke and ORNL FACE, respectively, according to the measurements (Figs2c,d, 5a,b). Most models simulated an initial (first year) increase in NPP as a result of eCO_2_ that was close to the observations. Notable exceptions were CABLE and CLM4, which systematically underestimated the initial response at both sites, as well as EALCO and ISAM, which overestimated the response for Duke FACE (Fig.5a,b). Nonetheless, no model simulated the underlying changes in *f*N_up_ and NUE correctly for both sites. At Duke Forest, according to the measurements, the increase in NPP was associated with a strong increase in *f*N_up_. The models generally underestimated the observed increase in *f*N_up_ and overestimated the increase in NUE. At ORNL, according to the measurements, the initial increase in NPP was associated with nearly equal increases of *f*N_up_ and NUE (Fig.5). Some models simulated a change in NUE in agreement with the observations (DAYCENT, GDAY, ISAM, LPJ-GUESS, OCN, TECO), but most models had a tendency to underestimate the increase in *f*N_up_.

The observed responses at the end of the experiment differed strongly between the two experiments (Fig.5c,d): the CO_2_ response of NPP at Duke Forest was maintained throughout the experiment, because the initial increase in *f*N_up_ was sustained with little change in whole-plant NUE. At ORNL, the CO_2_ response of NPP declined over time, because the initial increase in NUE declined as a result of higher allocation to N-rich fine roots. At the end of the experiment, NUE and *f*N_up_ were similar between ambient and elevated plots.

Most models showed signs of PNL (i.e. a progressively smaller enhancement in NPP as a result of N limitation) towards the end of the experiment at both sites (Fig.5c,d), but with varying strength and timing, causing an increasing spread among the models with the duration of the experiment. At Duke FACE, the models largely failed to capture the sustained NPP response to 11 yr of eCO_2_. The decline occurred despite increasing whole-plant NUE, because the models were not able to maintain an increased *f*N_up_ as observed (with the exception of ED2.1). At ORNL FACE, three of the 11 models correctly simulated the 10% decline in the initial response towards the end of the experiment (DAYCENT, LPJ-GUESS, SDGVM), and two models (GDAY, OCN) showed an even stronger decline, related to an early simulated onset of N limitation in the ambient treatment. Two models (ED2.1 and TECO) predicted an increase in the NPP response over time, fuelled by increases in plant N uptake, which were supported by a large pool of easily degradable SOM and inorganic N prescribed as initial conditions. Contrary to the observations, NUE and vegetation C : N strongly increased at ORNL in most models by the end of the experiment.

### Processes affecting NUE

#### N-based GPP and NPP

**Figure 6:**
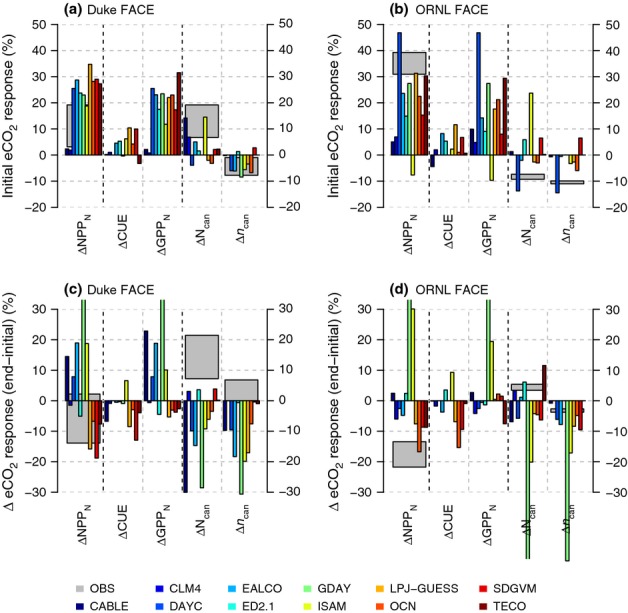
First year response of nitrogen (N)-based net primary production (NPP_N_) to elevated atmospheric [CO_2_] (eCO_2_) (a, b) and the change between the first year and the final 5 yr of the experiment (c, d) at the Duke and Oak Ridge National Laboratory (ORNL) Free-Air CO_2_ Enrichment (FACE) sites, respectively, as well as the response of plant carbon (C)-use efficiency (CUE), N-based gross primary production (GPP_N_) and canopy N, expressed as total canopy N (N_can_) and foliar N concentration (*n*_can_). The grey boxes denote the mean observed eCO_2_ response ± 1SE, where observations corresponding to model output are available.

Models differed strongly in their initial NPP_N_ response to eCO_2_ (Fig.6), generally overestimating the observed initial 11 ± 8% increase in NPP_N_ at Duke FACE and underestimating the observed 35 ± 4% increase at ORNL FACE. Although N limitation did not strongly affect GPP_N_ in the first year in most models, there were substantial differences in the first year's response among the models, in particular at ORNL FACE. Two models (CABLE and CLM4) showed an exceptionally low initial response of NPP at both sites (Fig.5). This low response was related to a near-zero response of GPP_N_ (Fig.6a,b). In CLM4, this response resulted from the assumption that plants down-regulate GPP directly when N limited: CO_2_ fertilization of GPP is calculated in the absence of N limitation, and then reduced using N-limitation scalars if *f*N_up_ is insufficient to support this amount of productivity. This low response did not happen in other models that followed a similar approach (DAYCENT and ED2.1), because of sufficient initial N supply. Another class of models simulated photosynthesis based on foliar N content (CABLE, GDAY, LPJ-GUESS, OCN, SDGVM, TECO). In these models, N limitation on GPP acts via foliar N concentrations: limited N availability reduces foliage N, which feeds back to limit GPP. This limitation takes time to develop, such that it was absent or weak in the initial response, but with a strong component of down-regulation in the longer term (Fig.6c,d).

Model predictions of the eCO_2_ effect on the other component of NPP_N_, CUE 1, can be readily categorized into three groups as follows:

1. models that assume that NPP is a fixed proportion of GPP (GDAY and DAYCENT) showed no change in CUE;
2. models that estimate *R*_a_ directly from biomass and temperature (CABLE, CLM4, EALCO, ED2.1, ISAM, LPJ-GUESS, SDGVM, OCN and TECO) predicted a transient increase in CUE, because the increase in respiration as a result of increased biomass lagged behind the immediate eCO_2_ effect on GPP. These models generally showed that CUE returned to its original value within the time course of the experiment (10 yr). In addition to these processes;
3. some models (CABLE, OCN) increased *R*_a_ under nutrient stress, when stoichiometric constraints prevented allocation of the assimilated C to growth.

For example, at ORNL FACE, CUE in OCN fell noticeably during the last years of the experiment (Fig.6d). This change was driven by a growing N limitation, which resulted in a build-up of labile C. Increased respiration was used as a mechanism to remove this excess accumulated C.

#### Whole-plant NUE

**Figure 7:**
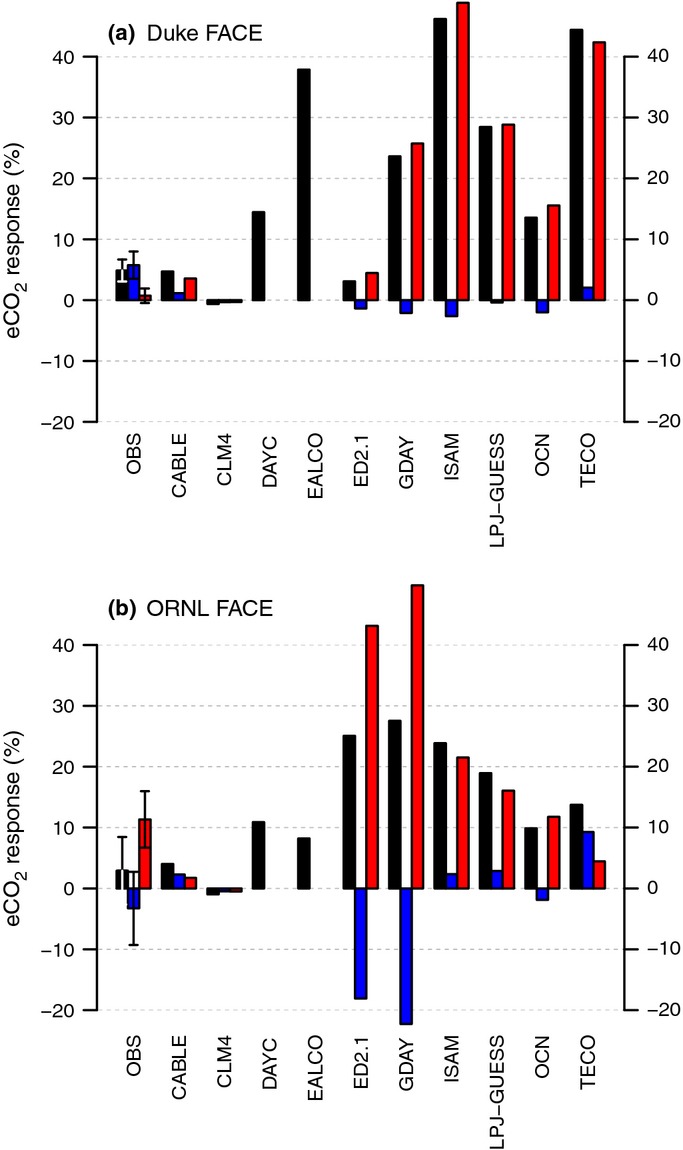
Change in nitrogen (N)-use efficiency of biomass production (NUE) at Duke (a) and Oak Ridge National Laboratory (ORNL) (b) Free-Air CO_2_ Enrichment (FACE) sites, integrated over the entire length of the experiment (1997–2005 and 1998–2008 for Duke and ORNL FACE, respectively). ΔNUE_alloc_ denotes the change in NUE attributed to changes in allocation to leaves, fine roots and wood, whereas ΔNUE_stoch_ denotes the change in NUE as a result of altered tissue C : N. The error bars denote ± 1SE. Black bars, ΔNUE; blue bars, ΔNUE_alloc_; red bars, ΔNUE_stoch_.

With eCO_2_, observed NUE at Duke Forest increased by 5 ± 2%, mainly because of a shift of allocation towards lower C : N tissue (wood), whereas the 4 ± 3% decline in foliar N had little effect on NUE (Fig.7). Despite the initially observed increase in NUE at ORNL FACE, NUE did not change over the course of the experiment (+2 ± 5%), as the effects of increased tissue C : N were compensated by increased allocation towards N-rich roots.

In the observations, the fraction of foliar N retranslocated before leaf shedding did not change significantly with eCO_2_ (−1.1 ± 0.4% at Duke Forest, 0.0 ± 14.3% at ORNL FACE), such that the retranslocation flux scaled with changes in total canopy N (see Fig.6). In most models (except EALCO), the retranslocation fraction did not vary with foliar N (or root N) content (Table1), such that, in agreement with observations, the retranslocation flux scaled with the total foliage (and root) N change. The effect of eCO_2_ on NUE can therefore be simply separated into its effects on stoichiometry and allocation (Fig.7) for those models that produced all of the variables required to perform these calculations. The model ensemble includes four alternative hypothesis combinations as to how whole-plant NUE changes with eCO_2_, namely:

1. assuming allocation and tissue stoichiometry to be constant (CLM4, TECO);
2. assuming flexible C : N ratios, but N-insensitive partitioning fractions (CABLE, GDAY, EALCO, SDGVM);
3. assuming constant tissue C : N ratios, but increasing root allocation with N stress (ED2.1); and
4. assuming the stoichiometry to be flexible and root allocation to increase with N stress (DAYCENT, ISAM, LPJ-GUESS, OCN).

Although the modelled NUE responses differed in magnitude among models, each model individually simulated similar trends at both sites, such that none of the models was able to simulate the observed difference in the NUE response between the sites, in particular, the observation-based interannual variability of the response at ORNL (Figs5). CABLE, which allows for the acclimation of tissue C : N only within narrow bounds, showed hardly any change in NUE, similar to CLM4, which simulates fixed tissue stoichiometry and allocation fractions (Fig.7). By contrast, models with a large flexibility in tissue stoichiometry (GDAY, LPJ-GUESS, OCN) consistently showed a stronger change in NUE as a result of increases in tissue C : N ratios rather than changes in allocation at both sites. The flexible C : N models showed a strong decline of foliar N at both sites, leading to a larger than observed decline in some models (Duke: CABLE, GDAY, LPJ-GUESS, OCN; ORNL: GDAY), which contributed to the excessive NUE response to eCO_2_ of these models.

The combined effect of the changes in allocation and stoichiometry in most models was that 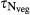 first declined, as a result of a greater growth of fast-overturning tissues (i.e. increased foliar growth as a result of increased NPP), but increased later in the experiment as tissue N concentration dropped and more N became incorporated into woody tissue. This model outcome is consistent with the observed response at Duke, but not ORNL FACE, where the strong increase in fine root growth resulted in a stronger decline in 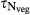 than suggested by the models.

In summary, models that include representations of flexible tissue stoichiometry, photosynthesis calculations based on prognostic foliar N and increasing fine root allocation under nutrient stress were generally more consistent with the observed trends of the component processes. However, because of difficulties in capturing the timing and magnitude of the response of stoichiometry and allocation (as well as the diverging predictions of plant N uptake; see next section on Processes affecting plant N uptake), these models did not appear to be generally superior to the other models considered here in terms of predicting the CO_2_ response of NPP.

### Processes affecting plant N uptake

As outlined in Materials and Methods a, changes in modelled *f*N_up_ can be attributed to: changes in the rate of net N mineralization (*f*N_min_), which depends on the total amount of SOM N (N_SOM_) and its turnover time (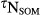); changes in the rate of depletion of the soil inorganic matter pool (ΔN_inorg_); and changes in NNE.

**Figure 8:**
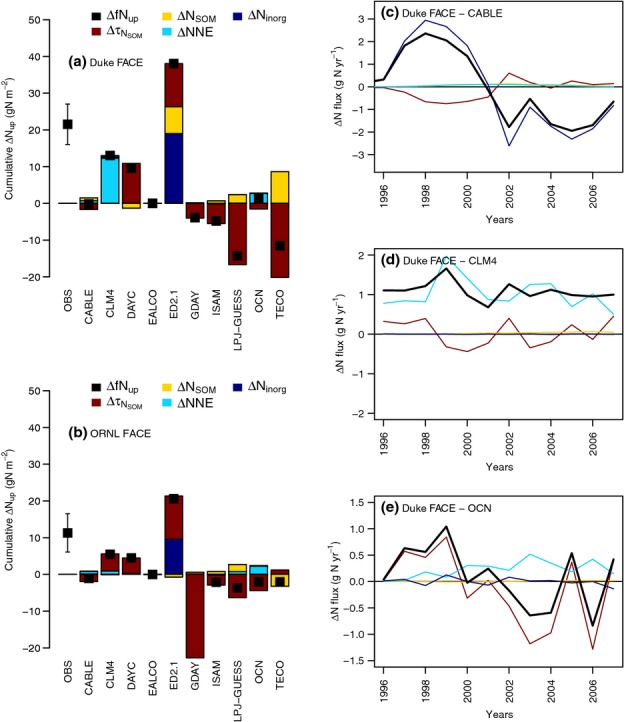
Cumulative plant nitrogen (N) uptake as a result of elevated atmospheric [CO_2_] (eCO_2_) over the length of the experiment, and its assignment to different mechanisms according to Eqns 4 and 5 at the Duke (a) and Oak Ridge National Laboratory (ORNL) (b) Free-Air CO_2_ Enrichment (FACE) sites. Positive values indicate an increase in plant N uptake, and negative values a decline. (c–e) Exemplary time courses of the net N balance for Duke forest, as predicted by CABLE (c), CLM4 (d) and OCN (e). Δ*f*N_up_, plant nitrogen uptake; 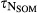, change in net N mineralization caused by a change in the soil organic N turnover time relative to the soil organic C turnover time; ΔN_SOM_, change in net N mineralization caused by a change in the organic N pool; ΔNNE, change in the ecosystem N balance (sum of N increases from biological N fixation and atmospheric N deposition and N losses to leaching and gaseous emissions); ΔN_inorg_, changes in the inorganic N pool. The error bars on the observations denote ± 1SE.

In SDGVM, *f*N_up_ was driven with observations and therefore this model is not considered further in this section. Among the other models, there are two alternative implementations of the processes that allow for a preferential increase in *f*N_up_ compared with microbial N immobilization under eCO_2_, leading to contrasting predictions (Fig.8a,b).

The first, employed by CLM4, is to increase the relative competitiveness of plants vs microbes for N. The plant's N demand is a function of potential GPP, which increases with eCO_2_. Conversely, the microbial N demand does not change strongly with eCO_2_, because CLM4 assumes fixed tissue C : N and therefore simulates no change in litter quality with eCO_2_, which would increase the N requirement of microbes and therefore immobilization. As a result, CLM4 showed a sustained increase in *f*N_up_ at Duke FACE, because less N was immobilized than under ambient conditions (Fig.8d).

The second mechanism is an emergent property of the CENTURY model (used by CABLE, DAYCENT, GDAY, LPJ-GUESS and OCN): initial increases in *f*N_up_ as a result of enhanced NPP lower soil inorganic N availability, which increases the C : N ratio of the newly formed SOM according to an empirical relationship. This reduces N immobilization during litter decomposition, as less N needs to be sequestered for the same amount of litter C transfer, increasing the availability of inorganic N for *f*N_up_ (Fig.8e). In most of these models, the increase was dampened or reversed within a few months or years because the models also apply a flexible tissue C : N. Increased N stress increased tissue (and therefore also litter) C : N ratios, leading to higher microbial N immobilization and therefore a reduction in the net N mineralization (*f*N_min_) to ambient or even below ambient rates, reflected as an increase in 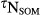, and therefore a decrease in the availability of inorganic N (Fig.8e).

A second factor affecting the eCO_2_ response of *f*N_up_ is the initial size of the inorganic N pool. Some models simulated an initial excess of inorganic N relative to plant N demand because of the site history (or the spin-up procedure; ED2.1, CABLE at Duke FACE and TECO at ORNL). An example is CABLE at Duke Forest (Fig.8c), in which the initial increase in *f*N_up_ was supported by the initially available inorganic N pool. This pool became exhausted after a few years of the experiment, leading to lower *f*N_up_ relative to the ambient plots in the later years of the experiment. The TECO model at ORNL had a much larger SOM pool, and with it gross N mineralization, than required by the forest's productivity, leading to a constant excess supply of N, which supported *f*N_up_ under eCO_2_.

The third factor is the ecosystem N balance (NNE), which depends on the rates of input via deposition and fixation, and the rates of loss via leaching and volatilization. A few models in the ensemble (CABLE, CLM4) simulated biological N fixation explicitly, but none suggested that eCO_2_ would alter fixation such that it would affect the net N balance. For the other models, the principal difference affecting total ecosystem N balance was whether the N losses were assumed to be proportional to the amount of N mineralized (CABLE, CLM4, GDAY, TECO) or whether they were a function of the simulated inorganic N concentration (CABLE, CLM4, EALCO, ISAM, LPJ-GUESS, OCN). In some of the models (CABLE, CLM4, DAYCENT, GDAY, LPJ-GUESS, OCN), ecosystem N losses were reduced, but the causal mechanism differed between the models: for example, GDAY, in which *f*N_up_ is assumed to be independent of plant N demand, and therefore eCO_2_, *f*N_min_ declined as a consequence of the higher microbial immobilization (higher litter C : N), which decreased directly the gaseous N losses in addition to reducing N leaching, because of lower soil inorganic N. In OCN, higher *f*N_up_ and increased N immobilization led to lower inorganic N, causing both lower gaseous and leaching losses.

In most models, the change in NNE was of the order of 1 g N m^−2^ over 10 yr. This reduction in N loss was not sufficient to prevent the onset of PNL in forests that take up 8.3 ± 0.4 g N m^−2^ yr^−1^, on average. The only exception to this pattern was the simulation of CLM4 at Duke FACE, where larger increases in *f*N_up_ substantially reduced gaseous N losses during autumn and winter, leading to a cumulative increase in *f*N_up_ of 12 g N m^−2^ (Fig.8a). Although this sustained increase avoided the progressive decline of *f*N_up_ in CLM4, it was not sufficient to explain the observed increase in vegetation N at Duke FACE.

### Time-integrated effect of eCO_2_ on ecosystem C and N

At Duke, *c*. 80% of the observed increase in cumulated NPP (3.1 ± 0.6 kg C m^−2^; 1997–2005) was sequestered in vegetation (2.5 ± 0.5 kg C m^−2^) and forest floor C (0.3 ± 0.1 kg C m^−2^), whereas soil C declined by *c*. 0.2 ± 0.1 kg C m^−2^ (Supporting Information Fig. S1). These changes were associated with increased vegetation N (12.2 ± 2.9 g N m^−2^), litter N (6.8 ± 2.6 g N m^−2^) and decreased soil N (25.0 ± 7.0 g N m^−2^). At ORNL, the observed enhancement of NPP (1.7 ±0.4 kg C m^−2^; 1998–2008) did not result in a significant change in biomass (0.0 ± 0.7 kg C m^−2^ and 1.2 ± 1.7 g N m^−2^, respectively), but soil C and N pools were increased slightly (0.2 ± 0.2 kg C m^−2^ and 11.5 ± 12.3 g N m^−2^, respectively).

Most of the models suggested that a large fraction of the NPP enhancement remained in vegetation C (Fig. S1), in agreement with the observed trends at Duke FACE, but in disagreement with those observed at ORNL FACE. Nevertheless, most models underestimated vegetation C sequestration at Duke FACE, because they underestimated the NPP enhancement and failed to predict the decline in SOM. Most models overestimated vegetation C sequestration in ORNL FACE, mostly related to failure in capturing accurately the allocation pattern and response (M. G. De Kauwe *et al*., unpublished; Fig. S1).

**Figure 9:**
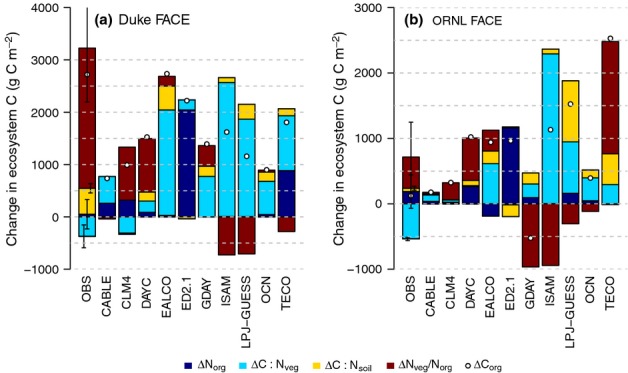
Total change in ecosystem carbon (ΔC_org_) as a result of elevated atmospheric [CO_2_] (eCO_2_) at the Duke (a) and Oak Ridge National Laboratory (ORNL) (b) Free-Air CO_2_ Enrichment (FACE) sites resulting from changes in the total organic ecosystem nitrogen (N) store (ΔN_org_), and vegetation and soil C : N ratios (ΔC : N_veg_ and ΔC : N_soil_), as well as changes in the fractionation of total ecosystem N between vegetation and soil, measured as the fraction of total ecosystem N in vegetation (*f*_veg_ = N_veg_/N_org_). The error bars denote ± 1SE.

The large observed increase in vegetation biomass at Duke Forest was supported mostly by a redistribution of N from soil to vegetation, as soil N stocks in the upper soil layers have probably declined over the course of the experiment (Fig.9a). However, there were significant differences in the magnitude of the transfer and vegetation C : N changes among the plots, causing large uncertainty in the attribution of the observed vegetation C increase. Although *f*N_up_ also increased in ORNL FACE, there was not a sustained increase in biomass N and C, because the rapid turnover of leaves and roots did not lead to a sustained increase in biomass N and C, which instead caused C and N sequestration in SOM (within the detection limit; Fig.9b). At both sites, bulk vegetation C : N decreased slightly with eCO_2_, despite the larger C : N in foliage, because of the larger contribution of foliage and root biomass to total biomass.

Consistent with the observations, increased organic ecosystem N (N_org_) played a minor role in most models (Fig.9). The exceptions of ED2.1 and TECO at Duke Forest were related to the assumed initial conditions (see section on Processes affecting plant N uptake). Changes in the ecosystem N balance, that is reduction in N losses, led to < 500 g C m^−2^ additional C sequestration (CLM4 and CABLE at Duke Forest; DAYC and LPJ-GUESS at ORNL FACE). Contrary to the observations, models that assume a flexible tissue C : N ratio (CABLE, EALCO, GDAY, LPJ-GUESS, OCN) predicted that a large fraction of the increase in ecosystem C storage at both sites as a result of eCO_2_ resulted from the increase in vegetation C : N ratios (see section on Processes affecting NUE). Only CLM4, which assumes fixed tissue stoichiometry, correctly predicted the decline in total vegetation C : N ratio at Duke Forest and the ensuing reduction in vegetation C storage capacity; this response resulted from the increase in foliar and root biomass. Changes in litter and soil C : N were generally of lesser importance in absolute terms, and roughly agreed with the observations. An exception to this was the projected large increase in litter C : N by LPJ-GUESS at ORNL FACE, associated with large litter fall of the deciduous trees and a strong decline in leaf N concentrations.

At Duke Forest, most models suggested that there was a net transfer of N to the vegetation (as a result of the increased *f*N_up_), which supported C accumulation in vegetation. However, the predicted increase was always less than half that observed. In LPJ-GUESS, the cumulative effect was a net transfer of N to the soil, probably related to the large fraction of C (and thus N) allocated to fast-overturning tissues (M. G. De Kauwe *et al*., unpublished). A net N transfer to vegetation initially also occurred in most models at ORNL FACE. However, in GDAY, LPJ-GUESS and OCN, the larger litter fall and the decreased litter C : N ratio at the deciduous site led to increased immobilization of N during decomposition. This provided a mechanism by which plant-available N became trapped in the SOM pool, effectively reducing the fraction of ecosystem N stored in vegetation, consistent with the PNL hypothesis.

## Discussion

The analyses presented here have separated the eCO_2_ response into time-dependent, observable components of the C and N cycle responses, which can be used to evaluate individual model processes and to identify key model weaknesses, as well as to identify the need for more observational constraints. The climate and N inputs, as well as the initial ambient levels of production, N uptake and NUE, were similar between the two sites, leading to the expectation that the different long-term trends in the eCO_2_ response of NPP and N uptake at Duke and ORNL FACE could be explained by processes associated with the different vegetation types encoded in the models. Despite the success of the models to simulate the initial eCO_2_ response of NPP at both sites, the models did not encode the relevant processes to explain the observed differences. Rather, most models followed the ORNL trajectory (progressively increasing N limitation) at both sites. In the following, we discuss the process representation of the most important C–N cycle linkages that contribute to the site and model–data differences.

### Model responses and underlying processes

#### Plant N uptake and net N mineralization

The increase in *f*N_up_ at Duke FACE was twice as large as that seen at ORNL FACE, in absolute terms and when integrated over the time of the experiment. This is a key factor in the observed, divergent NPP response at the two sites. The ensemble of models generally failed to simulate the magnitude of the observed increase in *f*N_up_ and the large difference between the sites, although some of the models possess mechanisms to increase root growth, and the specific N_inorg_ uptake capacity of roots or whole plants, under N stress. In most models, *f*N_up_ was tightly constrained by *f*N_min_, but only few ecosystem-scale observations are available for this quantity (Iversen *et al*., 2011). At ORNL FACE, the increased *f*N_up_ was probably related to the presence of plant-available N below the rooting zone of trees at the beginning of the experiment, resulting from past land use. Increased tree rooting depth and, probably, stimulation of SOM decomposition in these layers have added plant-accessible N (Iversen *et al*., 2008, 2011). The consideration of SOM depth profiles is missing in most ecosystem models, but this is likely to be relevant only under site conditions in which past land use determines the depth distribution of SOM. Increased microbial and fungal SOM decomposition following increased rhizodeposition (so called ‘priming’) is probably the cause of the large N transfer from soils to plants at Duke FACE (Drake *et al*., 2011); this is a further process not represented by the model ensemble. It is an open question whether this finding implies that models that do not incorporate such a mechanism must also have a low NPP response to gradually increasing atmospheric [CO_2_]. Under these conditions, the more gradual increase in plant N demand (Luo & Reynolds, 1999) might be satisfied by other mechanisms, such as the tightening of the ecosystem N balance or increased N fixation. Moreover, CENTURY-based models (DAYCENT, GDAY, OCN, LPJ-GUESS, TECO), which mimic the net transfer of N from soils to vegetation under increasing N stress, showed that the net N transfer based on N mining was limited. The pool of easily degradable N-rich material declined as a result of the increased N mining and declining litter quality, suggesting that ‘priming’ might be a temporary process relieving N stress.

#### NUE and ecosystem stoichiometry

The observed initial increase in whole-plant NUE was stronger at ORNL than at Duke Forest, and can largely be explained by the different magnitude of decline in foliar N concentrations and the diverging trends of total canopy N (Fig.6). The NUE enhancement decayed at ORNL FACE with increasing root allocation during the experiment, such that there was no strong change in NUE with eCO_2_ at both sites. The inclusion of flexible C : N stoichiometry, alongside increased below-ground allocation in response to eCO_2_ and increased plant N demand (M. G. De Kauwe *et al*., unpublished), appeared to be an important feature allowing the NUE response to CO_2_ to be captured because of the significant changes in foliar N concentrations. However, models that simulate flexible stoichiometry tended to overestimate the whole-plant NUE increase with eCO_2_. The probable reason for this overestimation is that the predicted changes in tissue C : N are not based on a hypothesis-driven prediction of C : N changes, but rather the emergent model outcome, as flexible stoichiometry in these models is the means to regulate C assimilation given plant-available N. Although the marginal change in photosynthetic capacity can be larger than the marginal change in foliar N (Friend *et al*., 1997), this does not seem to be sufficient to keep tissue C : N within the observed bounds, as shown by an exaggerated decline in foliar N concentrations at both sites. Other regulatory mechanisms, such as the acclimation of CUE under N stress, as implemented in the OCN model, can limit the reduction in tissue C : N ratios to variations within predefined bounds, but it is unclear whether such a mechanism exists in reality. Modelling approaches that maximize leaf photosynthetic gain given N and C availabilities may provide a more reliable framework to predict stoichiometric flexibility (Medlyn, 1996a; McMurtrie *et al*., 2008; Xu *et al*., 2012; McMurtrie & Dewar, 2013).

At both sites, the eCO_2_ effect on NPP_N_ according to the measurements initially increased (more so at ORNL than Duke FACE), but then declined to very low values of enhancement. In deciduous trees at both sites, this decline was not associated with a change in the relationship of photosynthetic biochemistry (*V*_cmax_, the maximum rate of carboxylation; *V*_jmax_, the maximum rate of electron transport at saturating irradiance) with leaf N (Norby *et al*., 2010; Ellsworth *et al*., 2011), whereas, at Duke Forest, older pine needles showed a reduced *V*_cmax_ per unit leaf N (Ellsworth *et al*., 2011). A number of models implement a leaf N dependence of photosynthetic biochemistry (Table1), and a few captured the overall trend in foliar N and GPP_N_. However, there was a large spread in the simulated eCO_2_ response of GPP_N_, both initially and in the longer term, despite the fact that (with the exception of DAYCENT) all models inherit the CO_2_ sensitivity of photosynthesis from the Farquhar model (Farquhar *et al*., 1980). As the effect of eCO_2_ on GPP_N_ is immediate, the uncertainty in the modelled initial GPP_N_ response is independent of the representation of N cycle feedbacks, and therefore not affected by the step increase in CO_2_. The differences among models were maintained when analysing daily data with a restricted range of meteorological parameters, instead of annually integrated values, a finding that excludes any difference caused by phenological biases (A. P. Walker *et al*., unpublished) which could also affect GPP_N_. The probable cause of these differences is alternative assumptions about the fraction of the canopy that is limited by light availability vs carboxylation rate, related to the canopy scaling of N and the depths of the canopy (Medlyn, 1996b). Varying stomatal responses to eCO_2_ may also have played a role (De Kauwe *et al*., 2013). Reducing this uncertainty requires a better representation of the changes in foliar N and the slope of the *V*_jmax_ : *V*_cmax_ relationship within the canopy and across different ecosystems (Maire *et al*., 2012). At the ecosystem level, alternative data sources, light response curves of net ecosystem exchange or GPP, derived from eddy covariance measurements, could facilitate the evaluation of the canopy-level light response across ecosystem types (Lasslop *et al*., 2010; Bonan *et al*., 2012).

#### Ecosystem N balance

Uncertainties in the observed changes in soil N stocks prevent any statistically meaningful assessment of whether eCO_2_ increased N capital as a result of changes in N inputs or outputs. Some models simulated increased plant N availability through reduced N losses from the ecosystem. Although these mechanisms added up to 12 g N m^−2^ (accumulated over the length of the experiment) in the most extreme case, they did not contribute strongly to the simulated C sequestration. Changes in the N balance may be an important factor in modelled eCO_2_ responses (Rastetter *et al*., 1997), but the effect was not very pronounced in the ensemble used in this study. None of these N loss reduction mechanisms was sufficient to explain the observations at Duke FACE. In agreement with previous observationally based studies (Drake *et al*., 2011), we conclude that a mechanism that increases plant N availability under plant N stress based on the enhanced mineralization of organic N is required for models to explain the observed trends at Duke.

### Limits of the observational constraints

The process inferences above rely on uncertain observations and implicit assumptions that require careful interpretation. The estimates of plant N uptake were inferred from the biomass production of plant tissues, their N concentrations and foliar N recovery on leaf shedding. Estimates of NPP and *f*N_up_ are therefore not independent, and so the estimated whole-plant NUE should be considered with caution. Increases in NPP without statistically significant changes in tissue N concentrations imply an increase in *f*N_up_, irrespective of whether the rhizospheric N uptake has indeed increased, or whether changes in foliar N retention (or perhaps labile amino acid reserves not accounted for in the observed tissue N concentration changes) have affected the N balance of the plants. This situation leads to uncertainty in the *f*N_up_ estimates for an individual year, and therefore the eCO_2_ response in the initial year of the experiment. However, the error associated with unaccounted for reserves diminishes when the estimates are integrated over time, and, on average, the translocation fractions did not change with time in the observations, further reducing the longer term error.

Uncertainty also results from the difficulties in measuring below-ground biomass and production, which is a fairly small contribution to total NPP at Duke Forest, but up to 40% of total NPP at ORNL under eCO_2_ (Iversen, 2009; McCarthy *et al*., 2010). Observations of fine root biomass should give suitably constrained estimates of the relative increase in root allocation under eCO_2_. However, uncertainty in the absolute below-ground C flux and, specifically, C flux to mycorrhizas propagates to uncertainty in annual NPP – and thus in the inferred N requirements to sustain the eCO_2_ response.

There is also substantial uncertainty in the observation-based estimates of net SOM changes with eCO_2_, resulting from a small signal-to-noise ratio and uncertainties in the sampling and analyses of the soil data (Jastrow *et al*., 2005). This uncertainty is primarily a result of the spatial variability of SOM, particularly for N (Iversen *et al*., 2012). The uncertainty in these measurements is sufficiently large to preclude reliable quantification of the net eCO_2_ effect on total soil and ecosystem C and N over the 10 yr of the experiment (Figs9, S1), as the expected change in SOM caused by CO_2_ is rather small. Therefore, the observations from Duke and ORNL Forests do not provide a robust constraint on the model N balance. Nonetheless, independent studies suggest that increased microbial decomposition may have resulted in a net transfer of N to vegetation at Duke FACE (Drake *et al*., 2011, 2013; Hofmockel *et al*., 2011), whereas increases in microbial activity with eCO_2_ may have been insufficient to compensate for the increased accumulation of N in SOM at ORNL FACE (Iversen *et al*., 2012).

Year-to-year variations in meteorological parameters influence both the ambient C and N cycling at the sites and the response to eCO_2_. These influences range from the direct effect of temperature on the CO_2_ sensitivity of photosynthesis (Hickler *et al*., 2008) to indirect effects resulting from interannual variations in the levels of drought stress (and thus eCO_2_–water-use efficiency interactions; De Kauwe *et al*., 2013) or N availability, following the sensitivity of SOM decomposition to soil temperature and moisture (Melillo *et al*., 2011). Assuming that the variability in the eCO_2_ response of NPP during the first 3 yr of the experiments was predominantly influenced by meteorological conditions, and not N availability (as suggested by most of the models), the weather-related standard error at Duke (1.3%) is lower than the across-ring variations (3%), whereas it is higher at ORNL (2.9% and 0.1%, respectively). These weather-related variations add uncertainty to our estimates of the initial response of NPP to eCO_2_, whereas they appear to be sufficiently small to allow us to decipher the long-term trend, which we assessed as a 5-yr mean towards the end of the experiment. We cannot rule out, however, that extreme events, such as the ice storm at Duke in December 2002 (McCarthy *et al*., 2007), have strongly altered the forest's C–N dynamics and thereby obscured the expected trajectory of NPP enhancement. Although the models' meteorological forcing contained these extreme events, none of the models incorporated the damage processes associated with, for instance, ice-break or wind damage.

A further complicating factor in the model–data analyses is that the magnitude of the N limitation of the CO_2_ response depends on various boundary conditions of the experiment, including the magnitude of the CO_2_ perturbation and the pool of plant-available N at the beginning of the experiment. The step increase in CO_2_ is much faster than projected future transient increases in atmospheric CO_2_. Thus, the experiment produces a suddenly increasing plant N demand (Luo & Reynolds, 1999) which could: (1) lead to an overestimate of the importance of nutrient constraints; and (2) trigger ecosystem processes that would not have occurred otherwise. The initial pool of easily plant-accessible N, either in the form of mineral N or readily decomposable dead organic material, is influenced by the land use history of the plots. It is difficult to estimate from bulk soil SOM measurements, as the net N mineralization depends on the partitioning of SOM into pools with different turnover times. In the absence of suitable initialization data, most models generated their initial condition based on site history, which caused uncertainty in the amount of net N mineralization, and thus N availability for plants, at the start of the experiment. Whether or not a model simulates PNL, and at what time scale, therefore depends not only on the model structure, but also on the initialization protocol. In particular, the ED2.1 model did not show signs of N limitation, because it did not simulate N inputs or losses; thus, the prescribed initial SOM pool provided ample inorganic N to support the growth of the trees throughout the simulation period. To minimize the effect of initial conditions, the models were evaluated in terms of the compatibility of their component processes with observations, rather than in terms of the average modelled productivity and N uptake response to CO_2_.

### Concluding remarks and recommendations for future experiments

The two FACE experiments initially showed a similar productivity response to eCO_2_, relative to a comparable baseline, in terms of forest productivity and forest N use, as well as climate and atmospheric N inputs. The long-term responses diverged strongly: the cumulated NPP response to eCO_2_ at the deciduous site was about half that of the evergreen site. The primary reason for this difference was that altered SOM dynamics increased plant N availability at Duke Forest at a rate that allowed the vegetation to maintain elevated levels of N uptake, whereas this did not happen at a sufficient rate at ORNL FACE. Furthermore, a corollary of the different allocation responses to eCO_2_ was that almost the entire NPP enhancement remained in vegetation biomass in Duke, whereas eCO_2_ did not alter vegetation biomass at ORNL FACE.

Many models in the ensemble were capable of reproducing the observed initial increase in NPP with eCO_2_. However, in the majority of cases, this response resulted from compensating errors in the underlying process responses, as the models did not correctly simulate the magnitude of the observed initial increase in plant N uptake at both sites, and wrongly attributed a large share of the increased NPP to enhanced NUE. This result cautions against a too simplistic model–data comparison and underlines the necessity of the detailed process-level evaluation. Comparing the process responses of ecosystem models against the observations provided essential information on model validity: we were able to identify component processes within particular models that were operating well (qualitatively and quantitatively), although the overall observed ecosystem eCO_2_ response was not accurately reproduced.

Models with flexible stoichiometry and allocation patterns that respond to N stress captured the qualitative responses observed at both sites. Ecosystem models with flexible tissue stoichiometry predicted a larger CO_2_ response of the NPP response despite a lower than observed CO_2_ response of *f*N_up_, and generally overestimated the observed increase in vegetation C : N ratio. Despite the conceptually increased accuracy of the results, this clearly shows that a more explicitly process-based approach to the modelling of stoichiometric flexibility is important for capturing the eCO_2_ response at these sites.

Despite the diversity of the modelling approaches employed here, all 11 combinations of C–N cycle processes include mechanisms consistent with the PNL hypothesis (Comins & McMurtrie, 1993; Luo *et al*., 2004), although the extent to which PNL was simulated varied depending on the assumed tightness of the stoichiometric constraint and the openness of the N cycle. Although this generally agrees with the observed trends at ORNL FACE, most models failed to simulate the sustained NPP enhancement at the Duke FACE site, because the mechanisms to increase N availability for plant growth included in these models are insufficient to explain the observed increases. This tendency to underestimate the net transfer of N from soils to vegetation under elevated CO_2_ at Duke calls for a better representation of below-ground processes, in particular root allocation and microbial responses to enhanced rhizodeposition.

Large uncertainty as to whether the observed changes in above-ground N stocks are caused by a redistribution of N from soils or to newly acquired N stems from the low signal-to-noise ratio in soil N inventories. Precise inventories well below the active rooting depth at the beginning of the experiment (as it may increase as the experiment progresses) would help, as would additional regular measurements of N balance components (N leaching and gaseous emission). Additional experiments using open-top chambers may further help to reduce uncertainty with respect to the below-ground mass balance and the net transfer of nutrients from soil to plants. Replicated factorial manipulation of nutrient availability and atmospheric [CO_2_] treatments could help to elucidate process interactions regarding allocation and stoichiometric responses to altered C and N availability. The strong increase in atmospheric CO_2_ might have triggered processes that would not have occurred if CO_2_ had increased at a more gradual pace. It would be of interest to investigate nutrient responses in ecosystem-level experiments, where CO_2_ is elevated more gradually to the maximum level in instalments, allowing the ecosystem to adjust at least partially to the new conditions. To reduce the dependence of the experimental results on the initial state of the ecosystem, it would also be desirable to conduct future elevated CO_2_ experiments with replication of different soil fertilities. This model comparison exercise has also underlined the increasingly recognized need for datasets from large-scale experiments to be collated into a central, versioned data repository that is readily accessible to modellers, if we are to fully capitalize on the potential for such experiments to inform models.

The different responses of several key processes at the two experimental sites, which cannot be explained by any of the models, imply that we should be sceptical of overarching statements concerning the responses of ecosystems to increasing levels of atmospheric CO_2_. There is currently insufficient knowledge to fully constrain the eCO_2_ response of global terrestrial ecosystem models, despite the existing body of experimental evidence. Nevertheless, the ecosystem models were able to capture important features of the experiments, lending some support to their projections (e.g. Thornton *et al*., 2009; Zaehle *et al*., 2010; Zhang *et al*., 2011).

## Appendices

**Table A1:** Overview of the models used and the representation of key processes in the carbon–nitrogen cycle. C, carbon; N, nitrogen; P, phosphorus; PFT, plant functional type; T, temperature; *f*(*x*), function of *x*.

		CABLE	CLM4	DAYCENT	EALCO
Key reference		Wang *et al*. (2010, 2011)	Thornton & Zimmermann (2007), Thornton *et al*. (2007)	Parton *et al*. (2010)	Wang *et al*. (2001)
Time step		30 min	30 min	1 d	30 min
Plant C acquisition	Assimilation (GPP)	Farquhar *et al*. (1980)	Collatz *et al*. (1991)	2 × NPP_act_	Farquhar *et al*. (1980)
	N dependence of gross photosynthesis	*f*(leaf N)	NPP_act_/NPP_pot_	None	*f*(leaf N)
	Autotrophic respiration	*f*(tissue N, *T*) + *f*(growth rate)	*f*(tissue C, *T*) + *f*(growth rate)	0.5 × GPP	*f*(tissue C, *T*) + *i*(growth rate)
	N dependence of whole-plant growth (if not GPP – *R*_a_)	None	Potential growth (NPP_pot_) limited by stoichiometric N requirement for new tissue growth	Potential growth (*f*(PAR, *T*, moisture, CO_2_)) limited by stoichiometric N requirement for new tissue growth	None
Plant N acquisition	Nitrogen fixation	Prescribed based on Wang & Houlton (2009)	*f*(NPP)	Plant-associated N fixation: *f*(N : P, plant N demand); soil N fixation: *f*(AET)	None
	Nitrogen uptake	*f*(plant N demand, soil N availability)	*f*(relative strength of plant and microbial N demand, inorganic N pool size)	*f*(root biomass, plant demand, soil N availability)	Competition of soil mineral N between plant and microbial
Plant growth	Allocation principle^1^	Fixed allocation fractions, which vary according to phenological state	Fixed allocation fractions, derived from observations at the sites	Hierarchical allocation factors, in which fine roots have priority over leaves and over wood, with prescribed maximum pool sizes	Fixed allocation fractions, which vary according to phenological state
	Maximum leaf area^1^	Prescribed (LAI = 8; excess C is allocated to wood and roots)	Predicted	Predicted	Prescribed from observations at the site
	N effect on allocation^1^	None	None	Nitrogen stress increases root allocation	None
	Plant tissue C : N stoichiometry	Flexible within 10% of the prescribed mean C : N	Fixed	Flexible within prescribed bounds	Flexible within prescribed bounds
Plant N turnover	N effect on turnover/mortality	None	Indirect via changes in NPP	Leaf turnover increases linearly with leaf N concentration	None
	N retention on leaf and root shedding	50% of leaf N, 10% of root N	Litter has a fixed C : N (PFT specific)	50% of leaf N	Retaining ratio depends on current tissue C : N ratio
Soil N turnover	SOM decay (other than dependent on soil *T* and moisture)	3 litter pools (metabolic, structural, coarse woody debris), 3 SOM pools with different turnover times, 1st order decay	3 litter pools, 4 SOM pools, all with different turnover times, 1st order decay	3 litter pools (above and below ground combined), 4 SOM pools, all with different turnover times, 1st order decay	3 litter pools; 4 SOM pools with different turnover rates, 1st order decay
	N effect on decomposition	Lignin : N ratio affects microbial efficiency and decomposition rate. Available soil mineral N constrains immobilization	Litter decomposition constrained by available soil N	Lignin : N ratio affects microbial efficiency and decomposition rate. Available soil mineral N constrains immobilization	Litter decomposition constrained by available soil N
	Soil C : N stoichiometry	Fixed for each pool	Fixed for each pool	*f*(mineral N concentration, within bounds)	*f*(mineral N concentration, within bounds)
Ecosystem N losses	N leaching	Proportional to mineral N pool	*f*(soil water N concentration, drainage)	DON + N leaching = *f*(precipitation, NO_3_ pool size)	*f*(mineral N concentration, drainage and surface runoff)
	gaseous N loss	Proportional to net N mineralization rate	Proportional to gross N mineralization + 10% of mineral N remaining in the soil	NO_*x*_, N_2_O, N_2_ fluxes, as a function of soil N pool size, temperature, water	None

		ED2.1	GDAY	ISAM	LPJ-GUESS
Key reference		Medvigy *et al*. (2009)	Comins & McMurtrie (1993)	Yang *et al*. (2009)	Smith *et al*. (2001, 2013)
Time step		15 min	1 d	30 min	1 d
Plant C acquisition	Assimilation	Farquhar *et al*. (1980)	Sands (1995, 1996)	Farquhar *et al*. (1980)	Collatz *et al*. (1991),*Haxeltine & Prentice* (1996)
	N dependence of gross photosynthesis	None	*f*(leaf N)	Stoichiometric downregulation of *V*_cmax_	*f*(leaf N)
	Autotrophic respiration	*f*(tissue C, *T*) + *f*(GPP)	0.5 × GPP	*f*(tissue N, *T*)	*f*(tissue N, *T*) + *f*(GPP)
	N dependence of whole-plant growth (if not GPP – *R*_a_)	Potential growth limited by stoichiometric N requirement for new tissue growth	None	None	None
Plant N acquisition	Nitrogen fixation	None	Prescribed	Predicted	Prescribed
	Nitrogen uptake	*f*(root biomass, plant N demand, soil N availability)	Fixed proportion of the inorganic N pool size	Michaelis–Menten kinetics, increases with increased plant N demand	*f*(plant N demand, soil *T*)
Plant growth	Allocation principle^1^	Functional relationships amongst leaf and sapwood (pipe-model), and sapwood and fine root biomass	Fixed allocation fractions, derived from observations at the sites	Dynamic allocation fractions, based on light, water and phenology	Functional relationships amongst leaf and sapwood (pipe-model), and leaf and fine root biomass
	Maximum leaf area^1^	Predicted	Predicted	Predicted	Predicted
	N effect on allocation^1^	Nitrogen stress decreases leaf : root ratio	None	None	Nitrogen stress decreases leaf : root ratio
	Plant C : N stoichiometry	Fixed	Flexible	Flexible within prescribed bounds	Flexible within prescribed bounds
Plant N turnover	N effect on turnover/mortality	Indirect via changes in NPP	None	Indirect via changes in NPP	Indirect via changes in NPP
	N retention on leaf and root shedding	50% of N is retained with leaf fall, but 0% with root turnover	50% of N is retained with leaf fall, but 0% with root turnover	Biome dependent	50% of N is retained
Soil N turnover	SOM decay (other than dependent on soil *T* and moisture)	Three SOM pools with varying turnover rates	4 litter pools (above/below metabolic and structural litter) and 3 SOM pools with varying turnover rates	4 litter/SOM above-ground pools, 4 litter/SOM below-ground pools and one inert organic matter pool with different turnover rates	5 litter pools (above/below metabolic and structural litter, plus an above CWD litter pool) and 5 SOM pools with varying turnover rates
	N effect on decomposition	Litter decomposition constrained by available soil N	Lignin : N ratio affects microbial efficiency and decomposition rate. Available soil mineral N constrains immobilization	Litter decomposition constrained by available soil N	Litter decomposition constrained by available soil N
	Soil C : N stoichiometry	Fast pool: function of mineral N. Slow and structural pool: fixed C : N	*f*(mineral N concentration, within bounds)	Fixed	*f*(mineral N concentration, within bounds)
Ecosystem N losses	N leaching	None	Fixed proportion of the inorganic N pool size	*f*(N pool size, drainage)	*f*(mineral N concentration, drainage)
	gaseous N loss	None	None	NH_3_ volatilization and denitrification losses	None

		OCN	SDGVM	TECO
Key reference		Zaehle & Friend (2010)	Woodward *et al*. (1995)	Weng & Luo (2009); updated
Time step		30 min	1 d	30 min
Plant C acquisition	Assimilation	Farquhar *et al*. (1980),*Kull & Kruijt* (1998)	Farquhar *et al*. (1980),*Harley et al*. (1992)	Farquhar *et al*. (1980)
	N dependence of gross photosynthesis	*f*(leaf N)	*f*(leaf N)	*f*(leaf N)
	Autotrophic respiration	*f*(tissue N) + *f*(growth rate) + excess respiration if labile C exceeds storage capacity, in the limits of the labile C pool size	*f*(tissue N, *T*)	*f*(leaf area, root and sapwood C)
	N dependence of whole-plant growth (if not GPP – *R*_a_)	*f*(labile C pool size, stoichiometric N requirement for new tissue growth)	None	Surplus C under N stress is allocated to woody biomass
Plant N acquisition	Nitrogen fixation	Prescribed	None	Prescribed
	Nitrogen uptake	Michaelis–Menten kinetics, proportional to root biomass, increases with increased plant N demand	*f*(soil organic C and N)	*f*(root C, plant N demand)
Plant growth	Allocation principle^1^	Functional relationships amongst leaf and sapwood (pipe-model), and leaf and fine root biomass	Leaf allocation determined as C balance of lowest LAI layer of the previous year. Root and wood allocation fixed fraction if GPP > 0	Resource limitations approach, prioritizing leaf over root and wood allocation
	Maximum leaf area^1^	Predicted	Predicted	Prescribed per plant functional type
	N effect on allocation^1^	Increased plant N demand increases root : leaf ratio	None	N limitation increases allocation to woody biomass
	Plant C : N stoichiometry	Flexible within prescribed bounds	Foliar N is prescribed from observations	Flexible within prescribed narrow bounds
Plant N turnover	N effect on turnover/mortality	Indirect via changes in NPP	None	None
	N retention on leaf and root shedding	50% of N is retained	None	50% of N is retained
Soil N turnover	SOM decay (other than dependent on soil *T* and moisture)	3 litter pools; 4 SOM pools with different turnover times, 1st order decay	4 litter pools, 4 SOM pools, with different turnover times, 1st order decay	5 SOM pools (metabolic litter, structural litter, fast SOM, slow SOM, and passive SOM) with different turnover rates, 1st order decay
	N effect on decomposition	Lignin : N ratio affects microbial efficiency and decomposition rate. Available soil mineral N constrains immobilization	n.a.	
	Soil C : N stoichiometry	*f*(mineral N concentration, within bounds)	Fixed	Flexible soil C : N ratios
Ecosystem N losses	N leaching	*f*(mineral N concentration, drainage)	None	*f*(mineralized N, runoff)
	gaseous N loss	*f*(mineral N concentration, soil *T*, moisture and respiration)	None	Fixed proportion of mineral N, regulated by soil *T*

1 See M. G. De Kauwe *et al*. (unpublished) for details.

## Appendix

**A2:** List of variable names used, as well as their description and unit. Tissue types considered are foliage (f), fine roots (r) and woody (w) biomass. C, carbon; N, nitrogen; DW, dry weight.

Variable	Description	Unit
*a*_*i*_	Fractional allocation to tissue type *i*	–
AET	actual evapotranspiration	mm yr^−1^
*B*_*i*_	Biomass of tissue type *i*	g DW m^−2^
C_org_	Ecosystem organic carbon	g C m^−2^
C_SOM_	Soil organic matter carbon (including the litter layer)	g C m^−2^
C_veg_	Vegetation carbon	g C m^−2^
CUE	Carbon-use efficiency (NPP/GPP)	– (g C g^−1^ C)
CWD	coarse woody debris	g C m^−2^
DON	dissolved organic nitrogen	g N m^−2^ yr^−1^
GPP	Area-based gross primary production	g C m^−2^ yr^−1^
GPP_N_	N-based gross primary production	g C g^−1^ N_can_ yr^−1^
*f*N_dep_	Atmospheric nitrogen deposition	g N m^−2^ yr^−1^
*f*N_fix_	Biological nitrogen fixation	g N m^−2^ yr^−1^
*f*N_*g*as_	Ecosystem loss of nitrogen through gaseous emission	g N m^−2^ yr^−1^
*f*N_leach_	Ecosystem loss of nitrogen through leaching	g N m^−2^ yr^−1^
*f*N_min_	Net nitrogen mineralization	g N m^−2^ yr^−1^
*f*N_up_	Plant nitrogen uptake	g N m^−2^ yr^−1^
*f*_trans_	Fraction of tissue N translocated before abscission	–
*f*_veg_	Fraction of organic ecosystem nitrogen in vegetation	–
LAI	leaf area index	m^2^ m^−2^
*n*_*i*_	Nitrogen concentration of tissue type *i*	g N g^−1^ DW
N_can_	Canopy nitrogen	g N m^−2^
N_org_	Ecosystem organic nitrogen	g N m^−2^
N_inorg_	Inorganic nitrogen in the ecosystem	g N m^−2^
n_som_	Soil organic matter nitrogen (including the litter layer)	g N m^−2^
N_veg_	Vegetation nitrogen	g N m^−2^
NNE	Net ecosystem nitrogen exchange	g N m^−2^ yr^−1^
NPP	Area-based net primary production	g C m^−2^ yr^−1^
NPP_N_	N-based net primary production	g C g^−1^ N_can_ yr^−1^
NUE	Nitrogen-use efficiency (NPP*/f*N_up_)	g C g^−1^ N
PAR	photosynthetically active radiation	μmol m^−2 ^ s^−1^
*R*_a_	Autotrophic respiration	g C m^−2^ yr^−1^
ρ_*i*_	tissue carbon density	g C g^−1^ DW
SOM	soil organic matter	g C|N m^−2^
	Turnover time of nitrogen in vegetation	yr^−1^
	Turnover time of nitrogen in soil organic matter (including the litter layer)	yr^−1^

## Supporting Information

**Fig. S1** Cumulative effect of elevated atmospheric [CO_2_] (eCO_2_) on carbon (C) and nitrogen (N) storage in the Duke and Oak Ridge National Laboratory (ORNL) Free-Air CO_2_ Enrichment (FACE) sites.
