# Supplementary material for: Evaluation of 11 terrestrial carbon–nitrogen cycle models against observations from two temperate Free-Air CO2 Enrichment studies
Source: New Phytol. 2014 Jan 28;202(3):803–22. doi: 10.1111/nph.12697 (PMC4288990; doi:10.1111/nph.12697)
Supplement: Supplementary file 1 — Fig. S1 Cumulative effect of elevated atmospheric [CO2] (eCO2) on carbon (C) and nitrogen (N) storage in the Duke and Oak Ridge National Laboratory (ORNL) Free-Air CO2 Enrichment (FACE) sites. [file nph0202-0803-sd1.pdf]

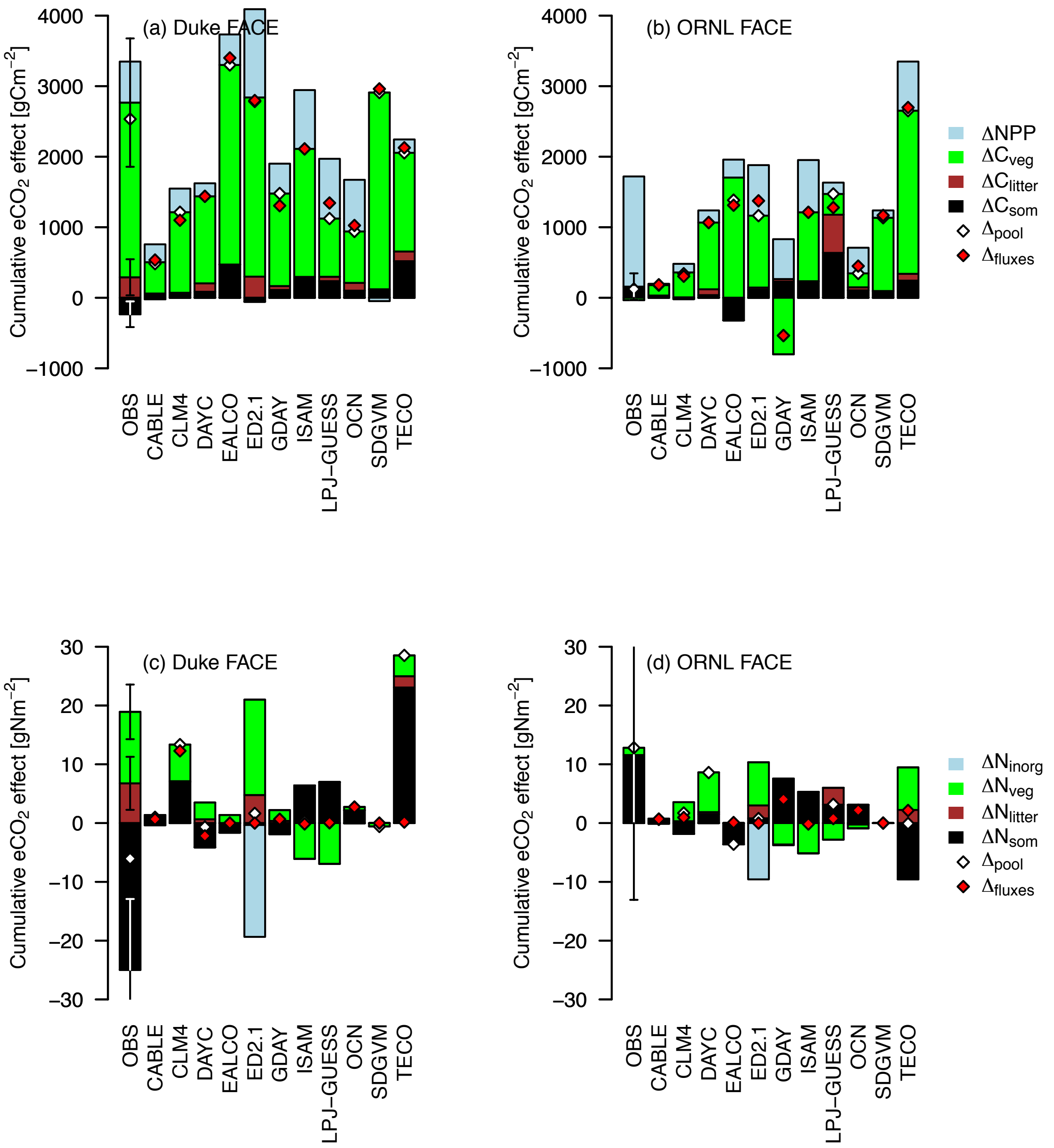

Figure S1: Cumulative effect of eCO_2 on C (a,b) and N (c,d) storage in the Duke (a,c) and ORNL (b,d) FACE sites. *NPP*: net primary production; N_{inorg}: inorganic soil N storage, $C|N_{veg}$: vegetation storage; $C|N_{litter}$: litter storage; $C|N_{soil}$: soil organic matter storage. Δpool|fluxes denote the total ecosystem mass balance based on cumulative changes in pool, or integrated net ecosystem C and N fluxes, respectively. The error bars denote ±1 standard error.
